# From Product Availability to Population Protection: Vaccine Deployment Readiness in Sierra Leone

**DOI:** 10.5334/aogh.5326

**Published:** 2026-09-07

**Authors:** Eric Nzirakaindi Ikoona, Lucy Namulemo, Rebecca Ikoona, Ronald Kaluya, Mohamed Alex Vandi, Foday Sahr

**Affiliations:** 1 National Public Health Agency, Freetown, Sierra Leone; 2 Uganda Counselling and Support Services, Kampala, Uganda; 3 Makerere University-Johns Hopkins Research Collaboration, Kampala, Uganda

**Keywords:** pandemic preparedness, vaccine deployment, institutional readiness, Sierra Leone, Ebola, COVID-19, mpox, global health security

## Abstract

*Background:* The 100 Days Mission has reorganised preparedness around vaccine development speed. Modelling suggests that meeting its target during the COVID-19 pandemic could have averted more than eight million deaths, conditional on equitable distribution. Distribution depends on a national pathway from authorisation through financing, cold chain, targeting, delivery, monitoring and community trust. The Mission measures speed at only one end of this system.

*Objective:* To develop and apply a country-anchored framework for the deployment-readiness layer of preparedness.

*Methods:* We conducted a comparative documentary analysis of three vaccine deployment events in Sierra Leone: COVID-19 vaccination in 2021, preventive Ebola vaccination in 2024 and mpox clade IIb vaccination in 2025. Sources included regulatory records, government and partner reports, national plans and aggregate programme records. We organised 11 established immunisation and emergency vaccination functions into a Deployment Readiness Chain. We traced constraints, handoffs, time to vaccination start and dated reach.

*Findings:* COVID-19 vaccination began 45 days after European Medicines Agency authorisation. Full coverage among people aged 12 years or older reached 73.2% by December 2022 and a reported 86.7% by October 2023. More than 84% of doses were delivered through surge campaigns or periodic intensification of routine immunisation. Nationwide preventive Ebola vaccination reached 17,454 of the 20,621 targeted workers, or 84.6%, in three weeks. It began 1851 days after authorisation, although a global stockpile had been in operation since 2021. Mpox vaccination began 106 days after outbreak confirmation and reached 186,053 people by 14 December through capabilities built during earlier emergencies.

*Conclusions:* For Ebola, the 1851-day interval spanned product authorisation, global preventive policy, financing and national activation. Product availability alone was not sufficient. Preparedness frameworks should evaluate deployment readiness alongside development speed. They should treat the global-to-national deployment pathway as one fundable category. Deployment readiness is built between emergencies, not during them.

## Introduction: Speed Measured at Only One End of the System

Since the 2021 global pandemic, preparedness has been organised around speed. The 100 Days Mission was articulated by the Coalition for Epidemic Preparedness Innovations (CEPI) and endorsed by the G7 and G20. It targets a safe, effective and authorised vaccine ready for initial manufacturing within 100 days of identifying a pandemic pathogen [1]. The empirical case for it is strong. Modelling estimated the value of meeting it during COVID-19 pandemic. Vaccines available within 100 days of the release of SARS-CoV-2 genome could have averted more than eight million excess deaths by the end of 2021 [2]. A peer-reviewed analysis put the figure for low- and middle-income countries alone at 4.8 million lives [3].

Both analyses carry the same condition. Their benefits depend on what happens after authorisation as much as on what happens before it. The projected gains assume improvements in manufacturing capacity and global distribution, and are substantially smaller without them [3]. The Mission’s authors have acknowledged that a second 100 days must follow [4]. It would focus on manufacturing scale, equitable distribution and delivery to the populations that need vaccines most.

That second period has not had a comparable anchor. Manufacturing has received attention through investment in regional production. Equitable distribution has been debated through the World Health Organization (WHO) Pandemic Agreement and through reflections on the COVID-19 Vaccines Global Access (COVAX) Facility [5, 6]. One layer has received far less analytical attention than development. That is the set of institutional conditions inside countries that determine whether an authorised, allocated and shipped vaccine reaches a person. This is not an argument against the 100 Days Mission. Development speed and deployment speed are complementary. The problem is that only one of them is measured.

COVID-19 showed what happens when the second layer is unready. Countries lacked regulatory frameworks to authorise novel products and cold-chain systems to move them. They lacked the workforce to administer them and systems to monitor coverage and adverse events [5]. Some countries returned or destroyed doses they could not deploy. Inequity in deployment compounded inequity in supply. By May 2022, 80% of people in high-income countries had received at least one dose, compared with 16% in low-income countries [7].

Sierra Leone offers an unusually clear case. Three vaccine deployment events occurred there within four years under three different conditions. COVID-19 vaccination began in March 2021 amid global scarcity. Nationwide preventive Ebola vaccination followed in December 2024, about five years after Ervebo was authorised [8–12]. Mpox clade IIb vaccination followed during an active outbreak in 2025 [13].

The Ebola case is the most informative because the vaccine was neither scarce nor new to the country. The 2014–2016 epidemic killed close to 4000 people in Sierra Leone, including nearly 7% of its health workforce [8]. Nearly 8000 Sierra Leonean frontline workers received the investigational platform in 2015, and licensed Ervebo vaccine was used in eight border districts in 2021 [14, 15]. A global stockpile had been in operation since 2021 [16]. Yet preventive policy was still evolving. Gavi opened its preventive programme in June 2024 after the WHO first recommended preventive vaccination for health and frontline workers in high-risk areas in May [9, 10]. Sierra Leone launched nationwide vaccination programme in December. The full 1851 days therefore cannot be assigned solely to national delay. It measures the time until policy, financing and country operations aligned. Product availability and prior use had not been sufficient.

We ask how vaccine deployment operates as a single institutional pathway, where its bottlenecks arise and how capability carries across threats. We propose the Deployment Readiness Chain, an 11-link, country-anchored framework connecting the global and national interface, national institutional architecture and measured deployment performance. It links the first 100 days of product development with the next 100 days of country deployment. The Joint External Evaluation (JEE) and the State Party Self-Assessment Annual Reporting tool (SPAR) provide broad International Health Regulations capacity evidence. The Vaccine Introduction Readiness Assessment Tool and Vaccine Readiness Assessment Framework (VIRAT/VRAF) and national deployment and vaccination plans add vaccine-specific detail [17–22]. The Chain connects these assessments to ownership, handoffs and dated performance. The next 100 days are real. It is institutional. It is fundable.

## Methods

### Design and case selection

We conducted a comparative documentary analysis. The unit of analysis was the deployment pathway for a defined vaccine event, not Sierra Leone as a whole. The three cases were selected purposively. Each represents a distinct condition: deployment under global scarcity, deployment after inter-emergency platform building and activation during a subsequent outbreak.

### Sources and selection

We reviewed regulatory records, WHO, Gavi and government reports, and legal instruments. We also reviewed Sierra Leone’s COVID-19 National Vaccine Deployment Plan, Joint External Evaluation and National Action Plan for Health Security. WHO readiness guidance and peer-reviewed and preprint campaign analyses completed the set [8–38]. The search ran through 30 July 2026. It combined Sierra Leone with COVID-19, Ebola or mpox, and added terms for authorisation, allocation, launch, coverage, readiness, logistics, workforce, surveillance and engagement. We checked reference lists and official sites.

Documents were included when they reported something dated or decided for one of the three events. This covered regulatory dates, allocation or procurement decisions, institutional functions, campaign milestones, target denominators and dated outcomes. Commentary without verifiable programme information was excluded. Public sources were supplemented by aggregate National Public Health Agency (NPHA) programme records, including Mpox Situation Reports 203 and 335.

The corresponding author extracted dates, outcomes and link-specific evidence into a case matrix. The author team reviewed the chronology and the classifications and resolved discrepancies by consensus. Regulatory records supported authorisation dates. Vaccination registers and aggregate reports supported counts and denominators. Legal instruments supported mandates. Government and partner reports supported programme context.

### Framework development and comparison

The 11 functions are drawn from Expanded Programme on Immunisation practices, emergency vaccination and WHO country-readiness guidance [17, 18]. We organised them as an end-to-end pathway with named handoffs. Their boundaries are analytic conveniences. Functions interact and may operate in parallel. Community trust, for example, affects targeting, delivery and monitoring, as well as demand. The contribution is the sequence, ownership and timing, not the invention of the functions.

For each case, we recorded whether each link was operating before or during the launch. We also recorded whether it caused a delay and whether it was later strengthened and reused. We assigned a principal bottleneck in two situations only. The first was where a source named the main constraint. The second was where the chronology showed deployment waiting on that function while the others were available. Where the record did not support a category, Table 2 presents the evidence without one.

We measured two distinct outcomes. Time to the start of vaccination was measured differently in each case. For COVID-19, it ran from European Medicines Agency (EMA) authorisation to the first national dose. For preventive Ebola, it ran from EMA authorisation to nationwide campaign start. For mpox, it ran from outbreak confirmation to operational vaccination start. Reach was the proportion of a stated target vaccinated by a stated date. Starting events differ between cases, so intervals are descriptive rather than a league table. Reach and start are reported separately throughout, because a fast start does not imply high reach.

### Ethics and positionality

Ethics committee approval and informed consent were not required. No identifiable data were analysed. Three authors hold National Public Health Agency roles. Their access supported the chronology but creates a risk of favourable interpretation. We addressed it through public-source triangulation, explicit uncertainty and no causal attribution to a single reform.

## The Deployment Readiness Chain

The Chain comprises 11 connected institutional functions. The first three connect global, regional and national actors. They are regulatory authorisation, financing and procurement, and allocation and shipment. The remaining eight operate mainly within national and sub-national institutions. They run from importation through cold chain, targeting, workforce, delivery, safety and coverage monitoring to community trust and demand. Readiness is the alignment of these functions across the whole pathway, not the strength of any one of them.

**Figure 1:**
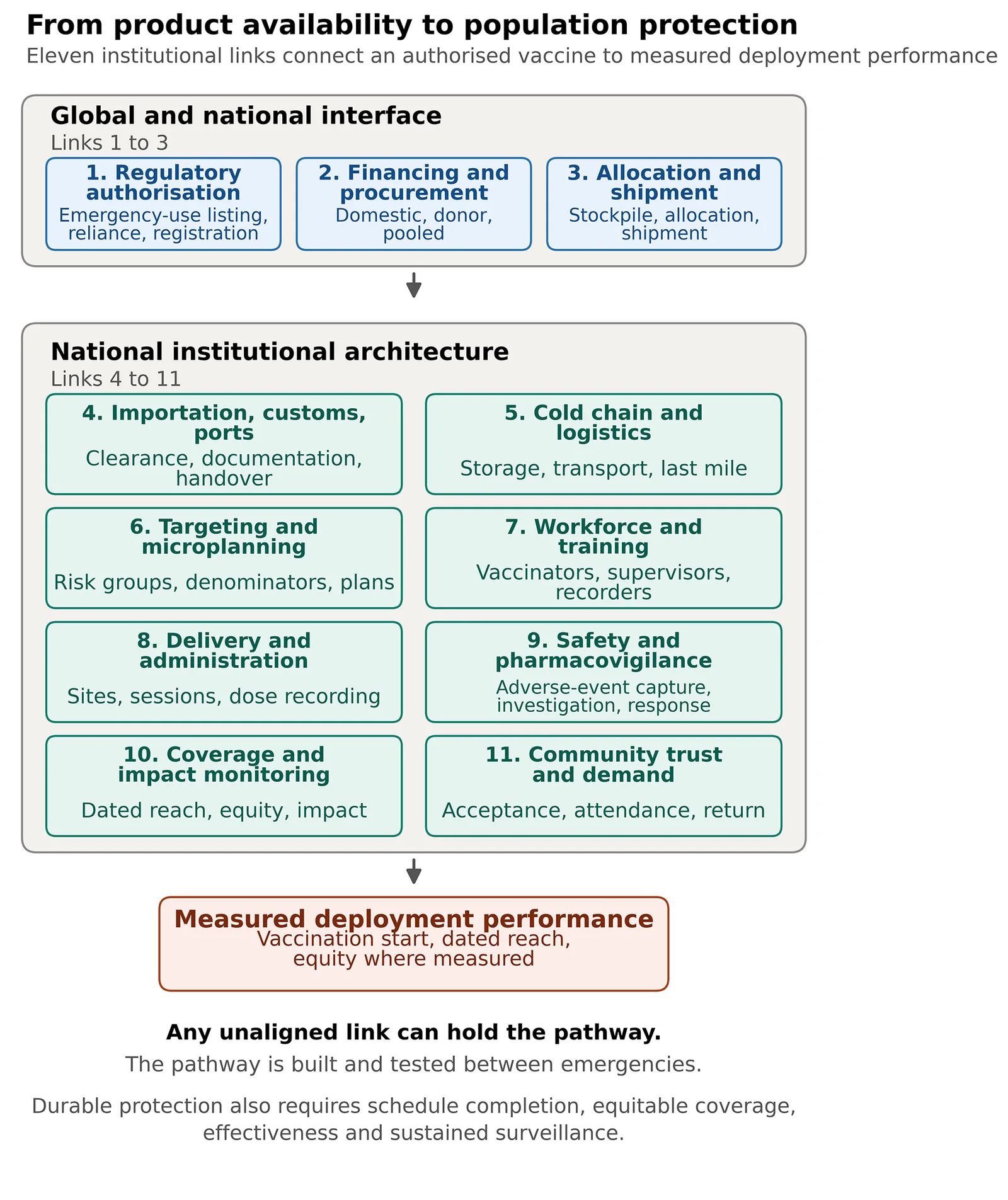
The 11-function Deployment Readiness Chain, from an authorised vaccine product to measured deployment performance. Functions 1 to 3 span the global and national interface; functions 4 to 11 operate nationally and subnationally. Any unaligned function can hold the pathway. The heuristic sequence derives from immunisation, emergency-vaccination and country-readiness practice. Durable protection also requires schedule completion, equity, effectiveness and surveillance. *Source:* Authors’ own figure.

Figure 1 presents the sequence and Table 1 defines each function, the evidence that traces it and the question it answers. Any link can bind. A country may have an authorised product, secured financing and a functioning cold chain system. It will still not vaccinate if targeting denominators are absent or if the population does not participate. This is why the pathway must be assessed as a connected system.

**Table 1:** Institutional functions and preparedness questions in the Deployment Readiness Chain.

NO.	FUNCTION	ROLE IN THE PATHWAY	TRACING EVIDENCE	PATHWAY QUESTION
1	Regulatory authorisation	National approval, registration or reliance pathway	Authorisation, registration or reliance record	Could the product legally enter national use?
2	Financing and procurement	Budget or procurement mechanism that secures doses	Budgets, financing commitments and procurement records	Were financing and procurement connected to secured supply?
3	Allocation and shipment	Rules and logistics for assigning and shipping doses	Allocation decisions, stockpile releases and shipping records	When did secured doses move towards the country?
4	Importation, customs or domestic handover	Clearance, documentation and receipt into the national system	Import approval, customs clearance and receipt records	When did doses enter national custody?
5	Cold chain and logistics	Storage, transport and last-mile distribution	Storage, transport and distribution records	Could doses reach delivery sites without interruption?
6	Targeting and microplanning	Risk groups, denominators, sites and session plans	Target denominators, site maps and session plans	Did a defined target become an operational plan?
7	Workforce mobilisation and training	Vaccinators, supervisors, recorders and logistics staff	Rosters, training and supervision records	Were trained teams available when and where needed?
8	Delivery and administration	Sites, sessions, dose administration and recording	Launch dates, session records and dose totals	When did vaccination start, and could delivery continue?
9	Safety monitoring and pharmacovigilance	Adverse-event capture, investigation and response	Adverse-event protocols, reports and investigation records	Could safety events be detected and managed?
10	Coverage and impact monitoring	Dated coverage, equity and impact assessment	Dated dose totals, denominators and stratified reports	What proportion was reached, by when and for whom?
11	Community trust and demand	Acceptance, attendance and return	Engagement plans and acceptance, attendance or rumour data	Did intended recipients accept and return for vaccination?

*Note:* The Joint External Evaluation and the State Party Self-Assessment Annual Reporting tool provide broad International Health Regulations capacity evidence. VIRAT/VRAF, national deployment and vaccination plans and programme records add vaccine-specific evidence. The functions are drawn from established Expanded Programme on Immunisation, emergency-vaccination and World Health Organization country-readiness practice. The Chain connects them through institutional responsibility, handoffs and dated performance.

Most of the work belongs between emergencies. Regulatory reliance pathways, financing mechanisms, cold-chain investment, workforce training, surveillance for targeting, pharmacovigilance and community engagement can all be built in advance and in parallel. During deployment, the same pathway traces activation. Durable protection requires more than the Chain delivers, including schedule completion, equitable coverage, effectiveness and sustained control.

## Results

### Case 1: COVID-19 vaccination, 2021

Sierra Leone received its first 96,000 AstraZeneca doses on 8 March 2021. They came through the COVAX Facility, within an initial allocation of 528,000 doses. The first national dose was administered on 15 March, 45 days after EMA authorisation, granted on 29 January [23–25]. Frontline workers and adults older than 70 years were vaccinated first, before eligibility widened. The binding constraint at this stage was global. Scarcity and phased allocation determined how much vaccine the country could use, not how fast it could start.

National capacity shaped what followed. Ebola-era regulatory pathways supported emergency authorisation of five COVID-19 vaccines. Average processing time fell from 42 days in March 2020 to 11 days by the end of 2021. Ultra-low-temperature equipment procured for Ebola met national COVID-19 needs, although facility-level gaps remained [25].

By 17 May, 63 days after launch, 62,132 people had received a first dose and 7427 had received a second dose [25]. These counts describe the early phase, not the programme outcome. Full coverage among people aged 12 years or older reached 73.2% by December 2022 and a reported 86.7% by 31 October 2023 [25]. These census-based estimates carry projection uncertainty. More than 84% of doses were delivered through 16 surge campaigns or periodic intensification. Surge teams expanded from 299 in the first three campaigns to 1385 in the final 13 [25]. The 45-day interval marks a start. Comparable reach took about two and a half years.

### Case 2: Nationwide preventive Ebola vaccination, 2024

Sierra Leone launched nationwide preventive Ebola vaccination on 5 December 2024 for frontline and high-risk workers across all 16 districts [8, 9]. It was the first campaign of its kind in West Africa. Target groups included health workers, traditional healers, community health workers, laboratory staff, motorcycle taxi riders and security personnel.

The 1851-day interval ran from EMA authorisation on 11 November 2019 to nationwide launch on 5 December 2024 [8–12]. It was not 1851 days of country delay. A global outbreak stockpile began in 2021. From 2021 to 2023, it shipped 145,690 doses, including 139,120 for high-risk-group prevention [16]. Sierra Leone had used the platform in 2015 and licensed Ervebo in eight districts in 2021 [14, 15]. Formal preventive policy came later. Gavi opened its preventive programme in June 2024 after the WHO first recommended preventive vaccination of health and frontline workers in high-risk areas in May [9, 10]. The nationwide campaign followed in December. The interval therefore spans global policy and financing as well as national activation.

By December 2024, the pathway had aligned. Global preventive policy and financing were available [9, 10]. The Public Health Act supported coordination, Pharmacy Board approval supported national use and the National Public Health Agency provided an institutional home [15, 26, 27]. Earlier investment in surveillance, logistics, field epidemiology and engagement supported targeting, delivery and demand [14, 15, 29–31, 37]. Launch came about six months after Gavi opened the programme.

The campaign reached 17,454 of 20,621 targeted workers, 84.6%, in three weeks [15]. Health workers accounted for 6058 recipients and security personnel for 5312, and the social worker target was met. Although 67.2% of recipients were male, women had higher adjusted odds of vaccination (1.51, 95% confidence interval 1.29–1.76) [15]. This is consistent with the sex composition of the targeted occupational groups. At launch, the Minister of Health framed the campaign as part of the national health security agenda and as protection for frontline workers [8]. It was presented as infrastructure rather than as a single event. The chronology supports a chain-wide institutional explanation. It does not establish that any single reform was sufficient on its own.

### Case 3: Mpox clade IIb vaccination, 2025

Sierra Leone confirmed its first mpox case on 10 January 2025 and declared a public health emergency [13, 36]. The response combined vaccination with surveillance, case investigation, contact tracing, laboratory expansion, infection prevention, risk communication and clinical care. Partners secured 61,300 initial doses for health workers and hotspot communities [33].

WHO reported a formal campaign launch on 20 March 2025 [33]. A later programme account identified 26 April as the operational start of vaccination, 106 days after confirmation, and we use that endpoint [34]. Subsequent allocations raised available doses to 283,500 and widened eligibility to contacts and other high-risk groups [34].

NPHA recorded 148,889 people vaccinated by 4 August against an operational target of 240,000, which is 62.0%, and 186,053 people vaccinated by 14 December [35, 38]. A Ministry report of 16 December stated that the outbreak had been declared over on 15 December [32]. December reports do not restate the August target. If that target still applied, the December total is 77.5% of it. The source counts, denominator, dates and qualifications are reproduced in Supplementary Tables S1 and S2.

The mpox interval begins with outbreak confirmation rather than authorisation, so it is not directly comparable with the other two. Its analytical value lies elsewhere. Programme reports link implementation directly to Ebola and COVID-19 experience. The same coordination structures, workforce, logistics and engagement networks were reused rather than rebuilt [32–34].

### Comparative findings

**Figure 2:**
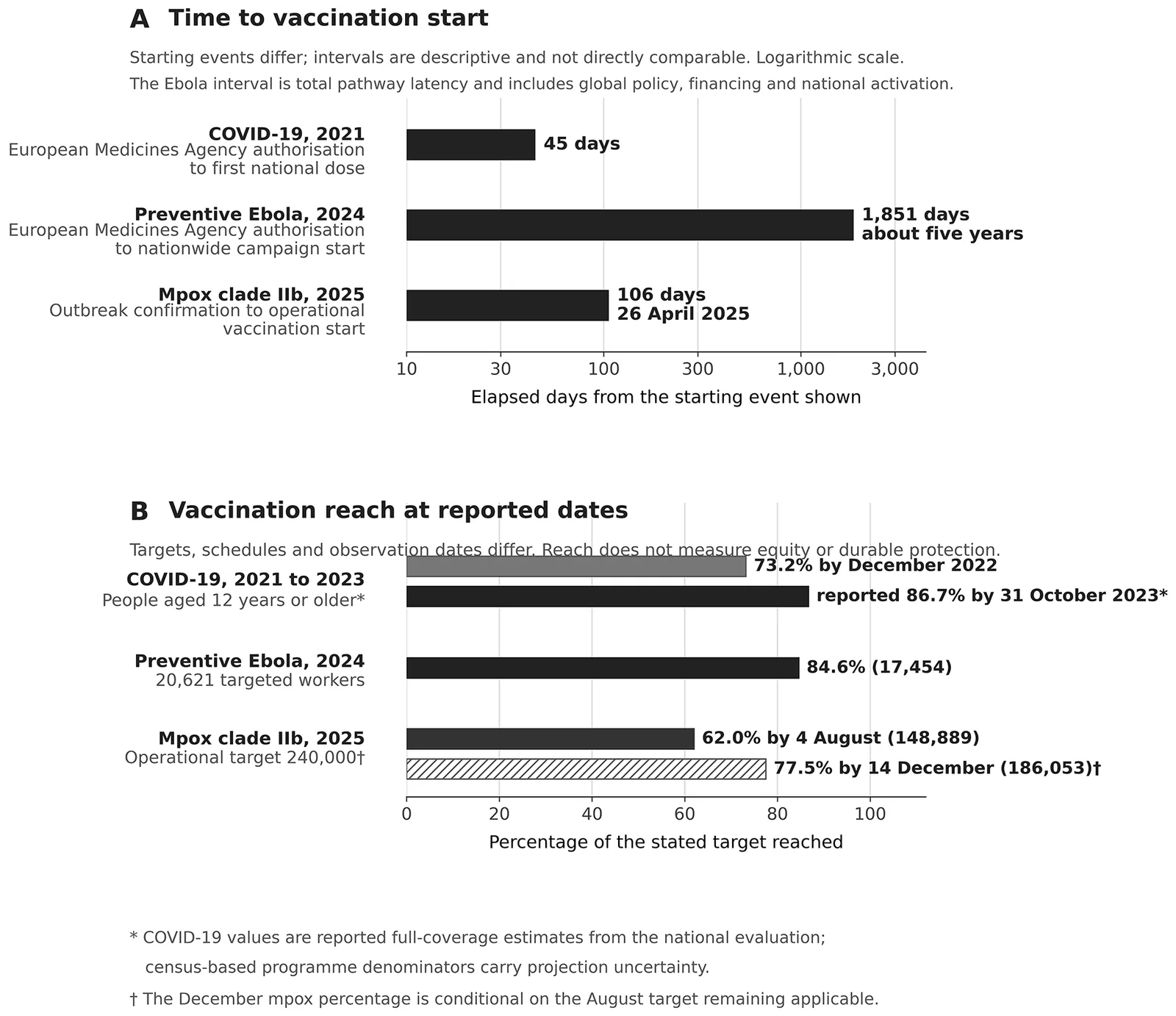
Time to vaccination start and reported vaccination reach for three deployment events in Sierra Leone, 2021–2025. Panel A shows descriptive intervals on a logarithmic scale. Starting events differ and are not directly comparable. Ebola’s 1,851 days include global policy, financing and national activation. Panel B shows dated reach. COVID-19 estimates carry denominator uncertainty. Ebola uses 20,621 targeted workers. Mpox uses an operational target of 240,000; its December percentage is conditional on that target remaining applicable. Reach does not measure equity or durable protection. *Source:* Cited records and aggregate National Public Health Agency reports; Authors’ own figure.

The three cases bind in different places. COVID-19 started in 45 days. Reported full coverage reached 86.7%, but took about two and a half years and relied chiefly on surge campaigns and periodic intensification. Preventive Ebola vaccination began 1851 days after authorisation. That total interval included global policy and financing as well as national activation. Once the pathway aligned, the campaign reached 84.6% of its target in three weeks. Mpox activated an existing platform and expanded reach over eight months. Figure 2 separates start from dated reach, while Table 2 compares the 11 functions across the three events.

**Table 2:** Link-by-link comparison of three vaccine deployment events. Principal bottleneck assignments were made by the author team, including authors with operational roles in the programmes, using the criteria stated in Methods section.

FUNCTION	COVID-19, 2021	PREVENTIVE EBOLA, 2024	MPOX CLADE IIB, 2025
1. Regulatory authorisation	Functioning: World Health Organization emergency-use reliance	Functioning by launch: Pharmacy Board product approval	Functioning: Pharmacy Board and national advisory approval
2. Financing and procurement	Functioning but externally dependent: COVAX Facility	Functioning: Gavi stockpile and partner financing	Functioning: global stockpile and partner financing
3. Allocation and shipment	Principal constraint: global scarcity and phased allocation	Functioning by launch: stockpile allocation secured	Functioning with expansion: 61,300 initial doses, 283,500 later available
4. Importation, customs or domestic handover	Functioning	Functioning	Functioning
5. Cold chain and logistics	Ebola-acquired ultra-low-temperature equipment reused; facility gaps remained	Functioning for the Ervebo campaign	Functioning for the national campaign
6. Targeting and microplanning	Functioning with phased age and risk eligibility	Functioning: occupational targeting across 16 districts	Functioning: contacts, health workers, hotspot and other high-risk groups
7. Workforce mobilisation and training	Surge teams expanded from 299 to 1385 across 16 campaigns	Functioning: national and district teams	Functioning: emergency workforce reused
8. Delivery and administration	First dose in 45 days; later delivery relied on 16 surge campaigns and periodic intensification	17,454 of 20,621 reached in three weeks	Formal launch reported in March; operational start at 106 days on 26 April; 186,053 vaccinated by 14 December
9. Safety monitoring and pharmacovigilance	Established during rollout; uneven reporting	Functioning campaign protocols	Functioning within the national response
10. Coverage and impact monitoring	73.2% full coverage by December 2022; reported estimate 86.7% by 31 October 2023	84.6% of target reached	186,053 people; 77.5% if the August target of 240,000 remained applicable
11. Community trust and demand	Active engagement; variable acceptance	Sustained engagement with occupational groups	Active outbreak drove mobilisation; community engagement expanded

*Note:* Categories are assigned only where the documentary record supports them. Starting events, endpoints and observation dates differ across cases.

Three findings follow. Any unaligned link can hold the pathway. A fast start does not guarantee rapid reach. Capability built for one threat can be reused for the next.

### Institutional change between the earlier and later events

Between 2019 and 2024, the record documents legal reform, the establishment of NPHA, workforce development, surveillance expansion, cold-chain investment and partnership coordination [15, 25–27, 29–31, 37]. Table 3 traces these enabling conditions and their use in the 2024 campaign. The mpox response shows the same capabilities used again for a different pathogen.

**Table 3:** Institutional enabling conditions across the study period.

ENABLING CONDITION AND RELEVANT CHAIN LINKS	DOCUMENTED POSITION, 2019–2021	DOCUMENTED POSITION OR USE BY DECEMBER 2024
Legal and institutional foundation (links 1 and 2, cross-cutting)	Public Health Ordinance of 1960; emergency functions distributed across Ministry directorates [26, 27].	Public Health Act in force; National Public Health Agency operating as the institutional home for public health emergency coordination [26, 27].
Targeting workforce (links 6–8)	Field epidemiology and district capacity expanding after Ebola [30, 31].	Field Epidemiology Training Programme cohorts and district teams supported national targeting and delivery [14].
Cold chain and logistics (link 5)	Ebola-acquired ultra-low-temperature equipment met national COVID-19 needs, although facility-level gaps remained [25].	Ervebo stored and distributed to campaign teams across all 16 districts [14].
Surveillance and microplanning (links 6 and 10)	Electronic surveillance expanded after Ebola; third-edition Integrated Disease Surveillance and Response guidance reached 1464 facilities by 2021 [30, 37].	The national electronic surveillance network and district teams supported targeting and microplanning [15, 37].
Community engagement (link 11)	Trust rebuilding after the 2014–2016 epidemic informed COVID-19 engagement [25, 30, 31].	Engagement involved health workers, traditional and religious leaders, transport workers and security forces [8, 15].
Partnership coordination (links 2 and 3, cross-cutting)	Coordination operated through Ministry and emergency-response structures [30, 31].	The Ministry, the National Public Health Agency, Gavi, the World Health Organization, the United Nations Children’s Fund and the International Coordinating Group on Vaccine Provision coordinated the national campaign [8, 9, 27].

*Note:* All abbreviations used in this table are written out in full at their first appearance in the text.

## Discussion

### Principal interpretation

Preparedness stops its clock too early. The 100 Days Mission target is not the problem. The absence of a matching country-facing clock is. Product availability is necessary. It is not sufficient. The interval between the two is institutional.

Sierra Leone demonstrates this three times. COVID-19 started in 45 days, but reported 86.7% full coverage took about two and a half years and repeated intensified delivery. Preventive Ebola vaccination began about five years after authorisation, then reached 84.6% of its target in three weeks. The interval included global policy and financing milestones, so it is total pathway latency rather than country delay alone. Mpox reached 186,053 people through a platform built for other diseases. The contrast makes the deployment gap visible without treating unlike intervals as a ranking.

Readiness is therefore a property of the connected system rather than of any capacity within it. An investment that strengthens one link while leaving the adjacent handoff untested buys less protection than its budget implies. The three cases support six claims about the deployment gap and about what closing it would require.

### First, the gap lay across the deployment pathway, not in product availability

Product scarcity alone does not explain the Ebola interval. Ervebo had been authorised since 2019. A funded stockpile operated from 2021, and Sierra Leone had prior experience [9, 14–16]. Global policy timing is a rival explanation. Gavi opened its preventive programme in June 2024 after the WHO first recommended preventive vaccination of health and frontline workers in high-risk areas in May [9, 10]. Sierra Leone launched nationwide vaccination in December. The 1851 days therefore span regulatory availability, global policy, financing and national activation. They are not 1851 days of national failure. The stronger finding is that product availability, stockpile access and prior use did not produce nationwide protection until the whole pathway aligned. COVID-19 was bound first by global allocation. Mpox shows what happens when a reusable country platform exists.

### Second, the pathway is reusable across threats

Successive versions of one national institutional platform carried three different products in four years. Coordination structures, workforce, logistics and engagement networks built for one threat were adapted for the next [15, 32–34]. Programme reports link the mpox response directly to Ebola and COVID-19 experience. Threat-specific investment underuses this property. Inter-emergency periods are when the platform is built. A platform built once can serve the threat that has not yet arrived.

### Third, urgency, population demand and international pressure act differently

The events faced different pressures. Urgency arose from active transmission. It was high during COVID-19 and mpox, but lower for preventive Ebola vaccination. Population demand was separate. It included willingness, social support and practical access. The WHO behavioural and social drivers framework distinguishes motivation from access barriers [39]. In rural Sierra Leone, mobile delivery and community mobilisation increased COVID-19 vaccination by about 26 percentage points within 48–72 hours [40]. Demand mattered, but the pathway still had to make vaccination reachable.

International pressure operated upstream. COVAX, scarcity and political attention shaped COVID-19 allocation [5, 23–25]. During mpox, international partners supported dose mobilisation and coordination [32–36]. Ebola drew on stockpile financing and partner support [8, 9, 15, 16], but lacked the acute mobilisation of an active outbreak. These forces are not interchangeable. Urgency compresses decisions. Demand affects acceptance, attendance and return. International pressure accelerates financing, allocation and coordination. None creates cold chain, workforce or target denominators. We therefore treat all three as contextual influences, not Chain functions.

### Fourth, deployment readiness can be measured

The 100 Days Mission has force partly because its target is a number. The next 100 days need comparable discipline. As further work, we propose two candidate measures. A deployment readiness score could assess the 11 links before an emergency. A deployment latency indicator could record days from a pre-specified starting event to first dose, then from first dose to dated reach. The starting event must be named because authorisation, recommendation, financing and outbreak confirmation are not interchangeable. Neither measure is specified or validated here. Both require development, weighting, piloting and external validation.

### Fifth, the chain nests inside existing readiness tools

JEE and SPAR assess International Health Regulations capacities across prevention, detection and response, including several on which vaccine deployment depends [20–22]. VIRAT/VRAF and national deployment and vaccination plans add vaccine-specific detail on targets, supply, cold chain, sites, workforce, safety, recording and demand [17–19]. Sierra Leone’s own COVID-19 National Vaccine Deployment Plan of October 2022 is a worked example [19]. It sets out coordination structures, prioritisation, cold-chain capacity and gaps, workforce requirements, adverse-event surveillance and data flows. These instruments are established, internationally accepted and already institutionalised in many countries. The Chain does not replace them.

What none of them does is time the whole pathway or name the institution that owns each handoff between one function and the next. A country can score well on capacity indicators and still wait years to deploy an authorised product. The indicators assess capacities separately rather than as a sequence. The Chain adds three things to what JEE and SPAR already produce. The first is the order in which functions must operate. The second is the institution accountable for each transition between them. The third is a dated measure of start and reach.

This makes integration straightforward. The 11 links can be populated inside an existing JEE or SPAR cycle rather than through a separate exercise. The JEE mission report supplies the capacity evidence. The Chain supplies the sequence, the ownership and the deadline. In practice, a country completing a JEE could add one page per link. Each page would record the lead institution, the alternate, the date the handoff was last tested and the time required to activate it. National action plans for health security would then carry deployment readiness as a costed line rather than as an implication.

### Sixth, deployment readiness should be funded and governed as one object

Preparedness finance enters countries through separate legal, surveillance, laboratory, workforce and emergency windows [28, 41–45]. The first Pandemic Fund call received requests above US$2.5 billion but awarded US$338 million [41]. The Chain provides one line of sight across these investments. Between emergencies, it identifies weak links, owners and costs. During a threat, it tests whether investment produced a timely start and reach. CEPI, the Pandemic Fund, Gavi and parties to the WHO Pandemic Agreement could use it to assess deployment readiness alongside development speed. Treating the links as one fundable category connects regulation, logistics, targeting, workforce, safety, monitoring and demand.

Governance should follow the same logic. Countries that have built and used deployment pathways across successive emergencies hold operational knowledge the global architecture rarely draws on. Consultation is not the same as a seat. National public health agencies are well placed to hold this work, because they connect surveillance, emergency coordination and evidence use. Responsibility remains shared with ministries of health, regulatory authorities, EPI programmes, finance ministries, district teams, communities and partners.

National capability cannot substitute for equitable global access. Regional manufacturing, pooled procurement and transparent allocation shorten upstream delay. Financing, regulation, logistics, workforce, monitoring and trust determine national use. Preparedness requires both, and the next 100 days are where they meet.

### Building the next 100 days between emergencies

Between emergencies, countries should populate all 11 links from JEE, SPAR, VIRAT/VRAF, national plans and programme records. Each link should be assigned a lead and an alternate institution. Regulatory reliance and emergency-use pathways, financing mechanisms, stockpile access and customs triggers should be pre-agreed rather than negotiated under pressure. Cold-chain routes, target denominators, microplans, workforce rosters, safety protocols, data flows and engagement networks should be kept current.

The complete pathway should then be tested at least annually, using tabletop and field simulation with a defined product and target population. The exercise should require every handoff. It should record the time from product availability to first dose, and from first dose to dated reach. It should end in a costed corrective plan with an owner, a financing source and a deadline. Gaps should enter national health security and EPI plans, emergency budgets and partner agreements, and be retested until closed.

## Limitations

This is a single-country analysis of three purposively selected events, drawing on sources of varying strength. Publication and document-selection bias are possible, and some aggregate records are not public. COVID-19 coverage estimates use census-based programme denominators and carry projection uncertainty. We therefore report the post-introduction evaluation’s estimate of 86.7% rather than treating it as a directly observed proportion [25]. The mpox operational target is an internal denominator that later reports do not restate. Its operational start date comes from a programme account rather than an official notice. Absolute counts are therefore more secure than the percentages.

Products, target populations, supply routes, urgency, starting events and follow-up periods differ across the three cases. The intervals are descriptive and are not a performance ranking. The study illustrates a framework rather than validating a scoring system. It cannot isolate individual reforms because they were undertaken as a coordinated programme. Three authors held operational or advisory roles in the institution being analysed. Their knowledge strengthened chronology and interpretation, but may also favour the institutional explanation. Triangulation reduces that risk but does not remove it. Transfer beyond Sierra Leone would require comparable EPI and emergency structures, legal authority, stockpile access, financing and subnational delivery capacity. The global recommendations should therefore be tested in other settings. Reach is not equity, effectiveness or durable protection, none of which this analysis measures.

## Conclusion

Preparedness cannot stop its clock at product availability. The Deployment Readiness Chain links the first 100 days of development to the next 100 days of deployment. It works with JEE, SPAR, VIRAT/VRAF and national plans to convert assessed capacity into owned handoffs and dated performance.

Sierra Leone did not deploy Ervebo in December 2024 because the vaccine was newly authorised. It deployed after global preventive policy, financing and country operations aligned. The same institutional platform then carried the mpox vaccine the following year in months rather than years. Product availability alone had not been enough. The pathway had to be ready.

The next 100 days are not a slogan. It is the institutional work that turns an authorised product into population protection. It can be measured. It can be funded. It is built between emergencies. It is the precondition for the first 100 days to save lives.

## Data Availability

Public documents are cited in the reference list. Mpox Situation Reports 203 and 335 remain under National Public Health Agency custodianship and are not publicly posted. The aggregate counts, denominators, dates and calculations used from those reports are reproduced in Supplementary Tables S1 and S2. No individual-level dataset was analysed. Additional access remains subject to agency approval and may be requested from the corresponding author.

## Additional File

The additional files for this article can be found as follows:

Supplementary File 1. Supplementary aggregate mpox tables.
