# Supplementary material for: From Product Availability to Population Protection: Vaccine Deployment Readiness in Sierra Leone
Source: Ann Glob Health. 2026 Sep 7;92(1):91. doi: 10.5334/aogh.5326 (PMC13564988; doi:10.5334/aogh.5326)
Supplement: Supplementary file 1 [file agh-92-1-5326-s1.pdf]

Supplementary aggregate mpox tables

From Product Availability to Population Protection: Vaccine Deployment Readiness in Sierra Leone

These tables reproduce the aggregate programme values used in the manuscript. They contain no individual-level or identifiable data.

Table S1. Dated mpox vaccination records

Report date	Source	Vaccinated count	Target stated in report	Reported or calculated reach
4 August 2025	NPHA Mpox Situation Report 203	148,889	240,000	62.0% (148,889 / 240,000)
14 December 2025	NPHA Mpox Situation Report 335	186,053	Not restated	77.5%, conditional on the August target remaining applicable

Table S2. Calculation and interpretation

Quantity	Calculation	Interpretation
August reach	148,889 / 240,000 x 100	62.0%
December reach	186,053 / 240,000 x 100	77.5% if the August operational target remained applicable
Increase in vaccinated count	186,053 - 148,889	37,164 additional recorded vaccinations

Qualification. Situation Report 335 did not restate the 240,000 operational target. The December percentage is therefore conditional. The absolute count of 186,053 is the more secure value.

Data custodianship. The source reports remain under National Public Health Agency custodianship. Further access is subject to agency approval and may be requested from the corresponding author at ikoonae@yahoo.com or eikoona@npha.gov.sl; telephone +232 76680085.
